# Comparative evaluation of antimicrobial efficacy of different combinations of calcium hydroxide against *Enterococcus faecalis*

**DOI:** 10.1186/s12903-023-03552-4

**Published:** 2023-11-11

**Authors:** Kavalipurapu Venkata Teja, Krishnamchari Janani, Kumar Chandan Srivastava, Deepti Shrivastava, Valentino Natoli, Marco Di Blasio, Macro Cicciu, Giuseppe Minervini

**Affiliations:** 1 Department of Conservative Dentistry and Endodontics, Saveetha Dental College & Hospitals, Saveetha University, Chennai, Tamilnadu India; 2 Department of Conservative Dentistry and Endodontics, SRM Dental College, Ramapuram, Chennai, Tamilnadu India; 3 Department of Oral & Maxillofacial Surgery & Diagnostic Sciences, College of Dentistry, Jouf University, 72345 Sakaka, Saudi Arabia; 4 Department of Preventive Dentistry, College of Dentistry, Jouf University, 72345 Sakaka, Saudi Arabia; 5 Department of Periodontics, Saveetha Dental College, Saveetha Institute of Medical and Technical Sciences, Saveetha University, Chennai, Tamil Nadu 602105 India; 6 Department of Dentistry, School of Biomedical and Health Sciences, European University of Madrid, 28670 Madrid, Spain; 7 Department of Medicine and Surgery, University Center of Dentistry, University of Parma, 43126 Parma, Italy; 8 Department of Biomedical and Surgical and Biomedical Sciences, Catania University, 95123 Catania, Italy; 9 Multidisciplinary Department of Medical-Surgical and Odontostomatological Specialties, University of Campania “Luigi Vanvitelli”, 80121 Naples, Italy; 10 Saveetha Dental College & Hospitals Saveetha Institute of Medical & Technical Sciences Saveetha University, Saavetha Dental College, Chennai, India

**Keywords:** Bioactive glass, Calcium hydroxide, Chitosan, Chlorhexidine, Silver nanoparticles, Microbiota, Endodontics

## Abstract

**Background:**

The study aims to compare the synergistic antibacterial efficacy of different combinations of calcium hydroxide as an intracanal medicament against *E. faecalis*.

**Material and methods:**

The current study included four hundred extracted human permanent mandibular premolar teeth. After complete chemo-mechanical preparation, the middle third of the root was sectioned using a rotary diamond disc and a total of 400 samples were obtained. The specimens were inoculated with *E. faecalis* for 21 days. After that, specimens were divided into five groups (*n* = 80) based on materials used for the disinfection of samples: Group I, calcium hydroxide alone; Group II, calcium hydroxide + 2% chlorhexidine gel; Group III, calcium hydroxide + 2% chitosan gel; Group IV, calcium hydroxide + 0.02% silver nanoparticle gel; Group V, calcium hydroxide + Bioactive glass S53P4. Dentin shavings from the apical third were obtained from the inner third of dentin were obtained using gates glidden no.1 to the apical depth, followed by no.2, 3, 4 and 5 analyzed for *E. faecalis* using the culture method. One-way analysis of variance (ANOVA) was used for data analysis, followed by post-hoc Tukey's test for multiple comparisons of means to check the difference in bacterial inhibition between the groups.

**Results:**

ANOVA results revealed a significant reduction of bacterial counts in all the groups compared (*p* < 0.001). Intergroup comparison showed maximum bacterial reduction *(p* < 0.001) with calcium hydroxide + bioactive glass S53P4 compared with other groups.

**Conclusion:**

Synergistic effect of calcium hydroxide showed better bacterial reduction compared to calcium hydroxide alone. Among the combinations evaluated, calcium hydroxide with bioactive glass, found to be most effective compared to other groups.

## Introduction

Optimal reduction of the microbial load from the root canal system is claimed to be the crucial step for the successful endodontic treatment [1, 2]. Studies have demonstrated 40–60% cultivable bacteria within the root canal system even after sodium hypochlorite irrigation [3–5]. Primarily, when the endodontic infections are considered, *E. feacalis* has shown to be the primitive bacterial cause in apical periodontitis cases. The claimed gram-positive microorganism is also shown to exhibit resistance and seen to be persistent in secondary root canal infections [2]. The typical nature of this microbe is its ability to form a mono-species biofilm in the root canal system, without any additional support from the other microbial species [6]. Even the *E. feacalis* has also shown higher resistance to the chemo-mechanical debridement and other adjunctive therapies rendered during the root canal treatment [7].

Various changes in the treatment procedures have been applied in Endodontics to overcome the above-mentioned issues. Although, the success of the endodontic treatment is still mainly dependent on the instrumentation and root canal disinfection [8]. It is essential to optimally disinfect the root canal system, to prevent the inherent tissue damage caused by bacteria and their byproducts released into the periapical areas [9]. The data from the current studies claim the inefficiency of conventional chemo-mechanical debridement in eliminating the microbial load from the root canal system [8–10].

Calcium hydroxide (CH) is the most popular intracanal medicament in Endodontics. Chemically it’s a strong base that is in contact with the aqueous liquids [11]. It exerts its antimicrobial action by dissociation reaction. Highly oxidant anions such as calcium and hydroxyl are released into the targeted site, which show extreme reactivity to various biomolecules [12]. The released hydroxyl ions, through direct or indirect contact, induce antibacterial effect through diffusion into the entire root canal system. Over the years, CH is considered a gold standard intracanal medicament [13, 14], but there is an ambiguity in its efficiency against *Enterococcus* species, mainly when colonized within the dentinal tubules [14]. *E. faecalis* is resistant to the alkaline pH which hinders the potentiality of the calcium hydroxide. *E. faecalis* tends to regulate its environmental pH by its proton pump property allowing its survival and perpetuating the infection. The previous report has revealed *E. faecalis* to be resistant and can adapt according to environmental change and penetrate the dentinal tubules. CH intracanal medicament is not equally effective for the bacterial types, inhabited in the root canal system. Moreover, the CH can be inactivated by dentin exudate evident from the periapical area [15–17].

Therefore, several studies focused on reporting the combination of medicaments to minimize the formation of resistant bacterial strain and eventually promote a synergistic effect with higher antibacterial efficacy [18–22]. Indeed, different agents, such as chlorhexidine [18], chitosan [19], silver nanoparticle [20], and bioactive glass [21], present a synergic antibacterial effect with CH. Chlorhexidine (CHX) has been claimed to be used as an potent intracanal medicament, both in vital and non-vital cases [22]. Furthermore, CHX intracanal medicament is claimed to be more effective than a CH when used as a dressing for eliminating *E. feacalis* [18]. Even though the mixture of CH and CHX is found to generate reactive oxygen species, the literature report stated no trace of parachloroaniline on the combination. This was mainly due to the immediate disintegration of CHX molecule which prevented the precipitate formation further [23].

Chitosan, a natural polysaccharide, is a copolymer of glucosamine and N-acetyl glucosamine. It has shown to be effective against a wide range of fungus and bacteria [24]. Previous studies were extensively conducted on chitosan as an intracanal medicament and tested its effectiveness towards various root canal pathogens [25, 26]. However, reports are lacking on the CH and chitosan combinations [19, 27], and testing its efficacy against the specific root canal pathogens.

Nanoparticles are nano-scale smaller sized molecules, with larger surface area and higher surface reactivity. Nanoparticles claimed to be alternative options in the modern dentistry, for both the prevention and the therapeutic benefit too [28, 29]. Silver nanoparticle (AGNP) has gained popularity in both restorative dentistry and endodontics as an additive in restorative materials [30, 31], as a dental adhesive [32], as an irrigant [33], intracanal medicament [34] and also as an additive antibacterial component of the root canal sealers [35]. Over the years, AGNP has been used as an intracanal medicament due to their strong antibacterial effect. They are claimed to be effective against gram-positive and negative bacteria [36] and multidrug-resistant bacteria [37]. Nevertheless, AGNP present some degree of toxicity depend on the concentration of the agent. Literature reported that these variations could be because of the differences in the size, dose, concentration, duration of action and the studied cell lines in various studies [38]. So, noteworthy is to study the critical AGNP concentration, alone or in combination with CH, which could induce a therapeutic benefit [20]. Hence, in the current study, we have planned to assess the real time benefit of combination of silver nanoparticles with CH as an intracanal medicament.

Bioactive glass (BAG) is a silica-based glass. The first-generation BAG is the 45S5. The composition of 45S5 is 45% SiO_2_, 24.5% CaO, 24.5% Na_2_O, 6% P_2_O_5_ [39]. The later developed compound with variation is the BAG S53P4. It is composed of SiO_2_ (53%), Na_2_O (23%), CaO (20%), P_2_O_4_ (4%) [40]. The BAG antibacterial property is dependent on the particle size. The smaller the size greater is the surface exposure of alkaline material to the liquid medium [41]. BAG has only been popular in recent days as an intracanal medicament. Its major advantage, when used as an intracanal medicament, is its antimicrobial ability along with an additional ion releasing capability, which helps in the alkalization of the medium [21]. There are several mechanisms explaining the mode of BAG action when used as an intracanal medicament. The accepted mechanism being the changes in the osmotic pressure, environmental pH, and needle like sharp debris produced by the materials, leads to the microbial cell damage, ultimately helping in the easier penetration of the antimicrobial components into the cytoplasm [42]. Although, growing evidence is in favor of using BAG as an intracanal medicament, with various studies focusing on its usage by altering the compositions and incorporating various chemical combinations.

Hence, the present study aimed to compare the synergistic antibacterial efficacy of different combinations of CH as an intracanal medicament against *E. faecalis*. The novelity of the current study lies in the different interventions namely CH, CHX, Chitosan, AGNP gel, and BAG 45S5 compared altogether. The null hypothesis states that there is no difference in the reduction of the *E.faecalis* count among the five different intracanal medicaments tested in the current study.

## Materials and methods

The present in vitro study was conducted after obtaining the ethical approval from the institutional ethical committee (IHEC/SDC/FACULTY/21/ENDO/133).

### Preparation of dentin specimen

Four hundred extracted mandibular premolars with mature apices were incorporated in the study. The inclusion criteria for the selected specimens were teeth with single root and single root canal, intact teeth with no caries, cracks or root resorptions. Teeth with root caries, curved canals and canal calcifications were excluded. The single canal morphology was confirmed with intraoral periapical radiographs taken at different angulations. The canal curvature was assessed using Schneider’s classification. Teeth with curvatures greater than 5° elicited through an intra-oral periapical radiograph, were excluded for the study. The initial apical diameter of the specimens was assessed by analyzing the CBCT sections. All the CBCT scanned specimens were assessed for their smallest diameter at 1 mm short of the root apex using an OnDemand 3D software (OnDemandedApp 1.0.9.2225; Cybermed, Inc, Seoul, South Korea) directly on axial sections, perpendicular to the canal. The entire assessment was done in an LCD monitor at (1366 × 768 pixels) resolution, to avoid selecting the specimens, whose diameter is more than the choosen preparation sizes in the current study. The current study protocol was similar to the previous literature [43–45].

An access preparation was performed with a high-speed round bur. After access cavity preparation, the patency of the canals was determined using K-files ISO # 10 (Dentsply Maillefer, Ballaigues, Switzerland). Only teeth whose canal width near the terminus was approx. comparable with ISO # 20 K-file were only included. After achieving the patency, the working length of the specimens was determined. After the working length confirmation, the instrumentation was carried out using protaper gold rotary files (Dentsply Maillefer, Ballaigues, Switzerland). The specimens were enlarged to a maximum size of 50/05 at the working length. In sue course of instrumentation, the irrigation was carried out using 10 ml of 3% sodium hypochlorite (Parcan, Septodont, India). A 30 guuage side vented needle irrigation (NaviTip, Ultradent Products, South Jordan, UT, USA) was carried out by placing the tip 1 mm short of the tentative working length. After the complete instrumentation, irrigation was carried out using 5 ml of 3% sodium hypochlorite and 3 ml of 17% ethylene diamine tertraacetic acid (MD Cleanser, Meta Biomed, India). The final rinse was carried out using saline and canals were dried using paper points.

After which, a rotary diamond disk was used to dissect the specimen to obtain 6 mm of the middle third of the root. The external diameter was standardized by removing the cementum from the root surface to 4 mm [46]. Then, using gates glidden drill no.3 (Mani Inc, Tochigi-ken, Japan), the internal diameter was standardized using a slow speed handpiece (NSK, Tokyo, Japan) till the apex. Finally, 3% sodium hypochlorite and 17% ethylene diamine tetraacetic acid were used for 5 min to remove the inoraganic and oragainc debris from the specimens. The final rinse was again carried out using saline and canals were dried using paper points. Next, the teeth were placed for 10 min in distilled water to remove the chemicals' remnant trace and then subjected to autoclave at 121 °C for 20 min [47].

### Contamination of specimen

The *E. faecalis* (ATCC 29212) strain was used as a test organism in the current study. It was grown on tryptone soya agar (TSB), suspended in 5 mL of TS broth and incubated for 4 h at 37 °C. The turbidity was equivalent to 0.5 McFarland standard. A 50 μL of the *E. faecalis* inoculums were transferred into pre-sterilized microcentrifuge tubes containing 1 mL of tryptone soy broth. The dentin specimens were transferred into the fresh broth containing *E. faecalis* at the end of 24 h and maintained under laminar flow (Clean Air, Mumbai, India). The culture purity was confirmed by sub-culturing 5 μL of the broth from the incubated dentin specimens in TSB on tryptone soy agar plates. The samples were incubated with *E. faecalis* at 37 °C for 21 days [47].

### Antimicrobial assessment

After 21 days of the incubation period, the specimens were rinsed with 5 mL of sterile water to remove the inoculated broth. Four hundred specimens were randomly allocated into 5 groups (*n* = 80) using a computer generated randomization procedure (www.random.org) and were assigned to the groups, based on the different intracanal medicament as follow:

- Group I: CH (Ref no.23,923–2; SigmaAldrich, Mumbai, India);
- Group II: CH + 2% CHX gel (Ref no.24800; Sigma-Aldrich);
- Group III: CH + 2% chitosan gel (Everest Biotic, Bangalore, India);
- Group IV: CH + 0.02% AGNP gel (huzheng nanotechnology co ltd);
- Group V: CH + BAG S53P4 (AbminDent; Abmin Technologies Ltd, Turku, Finland).

The particle size for BAG S53P4 was 45 µm. For Group II, III, IV and V, sterile saline was used to mix the agents and adjust to a ratio of 1.5:1 to obtain paste-like consistency. The medicament in group 1 was mixed with sterile saline at a ratio of 1.5:1 [48]. The reason for adjusting the ratio was to standardaize the obtained mix as suggested in the previous similar study design [48]. Hence, in the current study, the agents were mixed and adjusted to a ratio of 1:5:1. The medicaments were then placed into the root canal, after which the ends of the specimen were sealed with temporary cement (cavit G;3 M ESPE, Seefeld, Germany) [47]. The samples were incubated in an anaerobic environment at 37 °C for seven days.

After seven days, the coronal seal was removed and the medicaments from canals were taken out using distilled water. Dentin shavings obtained from the inner third of dentin were obtained using gates glidden no.1 to the apical depth, followed by no. 2, 3, 4 and 5 [46, 47, 49, 50]. Before the experimentation, the initial weight of the uncapped Eppendorf tube was recorded in triplicate using an analytical balance (Sartorius Intec, Hamburg, Germany). The tubes were then finally incubated at 70 degree celcius for 5 days. The postinstrumentation dentin shavings were then weighed. Finally the dentinal shavings were immersed in 1 ml of TSA broth in 1.5 ml Eppendorf tubes. The tubes were vibrated in Fisher Vortex equipment for 2 min to homogenize the samples.

### Culture methods for colony-forming units

A tenfold dilutions were prepared, and 1 ml of aliquots of suspensions were seeded on a Petri dish with 5% sheep blood TSA plates and then incubated at 37 °C for 48 h. The colony-forming units (CFU) grown were counted using a stereomicroscope, and log transformation was carried out for the values obtained.

### Sample size and statistical analysis

A priori sample size calculation was done using G* power software (Universität Düsseldorf: Psychologie – HHU). Considering the ANOVA test (main effects and interactions) for comparing means of five study groups with an effect size (Cohen’s d value) of 0.25, confidence interval (1-β error) of 95% and 0.05 α, a total sample of 400 was calculated. With this sample size, the power of the study was estimated to be 0.95. Each study group has a sample size of 80. The data were statistically analyzed with a one-way analysis of variance (ANOVA), followed by post hoc Tukey's HSD multiple comparisons of means to check the difference in bacterial inhibition between the groups.

## Results

**Fig. 1:**
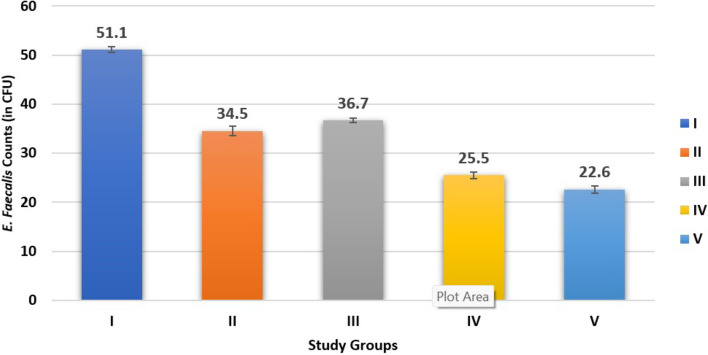
Bar graph showing Intergroup comparative analysis of *E. faecalis* Counts

The mean CFU for all the groups were Group I = 51.23 × 10^2^, Group II = 35.23 × 10^2^, Group III = 37.26 × 10^2^, Group IV = 26.14 × 10^2^, Group V = 23.25 × 10^2^. ANOVA results revealed the statistical reduction (p < 0.001) in all the groups compared (Fig. 1). Intergroup comparison revealed that Group V was most effective (p < 0.001) against *E. faecalis* followed by Group IV, Group II and Group III. The least effective was Group I where CH was alone. The current study results have shown CH combinations to be more effective against *E. feacalis* as compared to the CH alone. Among the various combinations assessed, CH + BAG S53P4 combination has shown more reduction in the CFU counts after the usage of medicament. Whereas the CH + chitosan combintion was the least effective in reducing the CFU counts among all the combinations compared (Table 1).

**Table 1:** Tukey’s Post-hoc analysis showing intergroup comparison of *E. faecalis* counts

Study Group		95% Confidence Interval Value		
		Lower Bound		Upper Bound
I	II	< 0.001^¶^	15.70	17.50
	III	< 0.001^¶^	13.50	15.30
	IV	< 0.001^¶^	24.70	26.50
	V	< 0.001^¶^	27.60	29.40
II	I	< 0.001^¶^	-17.50	-15.70
	III	< 0.001^¶^	-3.10	-1.30
	IV	< 0.001^¶^	8.10	9.90
	V	< 0.001^¶^	11.00	12.80
III	I	< 0.001^¶^	-15.30	-13.50
	II	< 0.001^¶^	1.30	3.10
	IV	< 0.001^¶^	10.30	12.10
	V	< 0.001^¶^	13.20	15.00
IV	I	< 0.001^¶^	-26.50	-24.70
	II	< 0.001^¶^	-9.90	-8.10
	III	< 0.001^¶^	-12.10	-10.30
	V	< 0.001^¶^	2.00	3.80
V	I	< 0.001^¶^	-29.40	-27.60
	II	< 0.001^¶^	-12.80	-11.00
	III	< 0.001^¶^	-15.00	-13.20
	IV	< 0.001^¶^	-3.80	-2.00

Group I—Calcium hydroxide (CH); Group II—CH + 2% chlorhexidine (CHX) gel; Group III—CH + 2% chitosan gel; Group IV—CH + 0.02% silver nanoparticle (AGNP) gel; Group V—CH + Bioactive glass (BAG) S53P4. Results are expressed in mean ± Standard deviation; CFU- Colony forming Units; ⁋ *p* < 0.001

## Discussion

Endodontic treatment should focus on the elimination of bacterial biofilm from the root canal system [51]. Habitation of bacterial biofilm within the canal is the primary cause of pulpal and periapical disease [52]. Incomplete removal of these microorganisms during the endodontic treatment can result in infection, which tends to be majorly responsible for root canal treatment failures [53, 54]. Although, *E. faecalis*, is one of the most prevalent species found in canals of teeth with post treatment apical periodontitis, the current eveidence suggests that the post treamnet infections are usually mixed and caused by diufferent bacterial combinations [55].

Moreover, the root canal system's dentin composition and anatomical complexities in persistent infection tend to cause limitation to root canal disinfection [56]. In such a situation, chemical disinfection alone might not be sufficient for the complete elimination of microorganisms. Therefore, the choice of intracanal medicament plays a significant role in this condition. Haapasalo and Ørstavik proposed an in vitro model for the assessment of disinfection of dentinal tubules [57]. A modified model was used in the present study as we obtained the extracted teeth from humans compared to the previous ones, which used bovine teeth [46].

The present study intended to assess different agents in combination with CH when used as intraoperative root canal medicament. There are no reports on CH with BAG and CH with AGNP. The literature is not as specific on its usage, especially as an intracanal medicament or an inter-appointment dressing controlling the endodontic infections. As compared to the CH intracanal medicament, BAG is claimed to be superior in terms of its remineralisation ability and helpful in bone formation by hydoroxyapatite layer formation [21]. Hence, studies are more warranted on assessing the various benefits on using BAG as an intracanal medicament. As other medicaments, it also needs as an adequate vehicle for easier mixing and dispensing the medicament into the root canal system. The vehicle used for intracanal medicament, should also support for ion dissolution, solubility and reabsorption. Distilled water and saline are the ideal vehicles for BAG when used as an intracanal medicament [58]. The literature evidence from the *ex-vivo* and in vitro studies is supportive for the its immense biocompatibility, osseo-induction and osseo-conduction ability [59]. The results of the present study have revealed 23.25 × 10^2^ bacterial reduction with CH + BAG S53P4 followed by 26.14 × 10^2^ bacterial reduction with CH + 0.02% AGNP gel. However, CH + 2% CHX gel and CH + 2% chitosan gel had an overall reduction of only 35.23 × 10^2^ and 37.26 × 10^2^ respectively. Therefore, the hypothesis null was rejected since there was a statistical differences between the groups. In addition, the results revealed the most negligible bacterial reduction when CH was used alone, without any combinations.

BAG S53P4 has shown significant bacterial reduction. However, the mechanism of action is not fully understood. The possible activity might be due to the pH increase and osmotic pressure of the aqueous BAG that cause surface deterioration due to Na, Si, PO_4_ ions, which eventually causes the death of microorganisms [40, 60].

Studies have also shown the particle size of BAG can contribute to surface deterioration [41]. BAG S53P4 has a particle size of 45 µm. Therefore, this can also be a reason for good antibacterial activity.

The first study by Zehnder et al. assessed the antibacterial efficacy of BAG S53P4 compared with CH and reported CH as ineffective compared with a BAG [40]. On the other hand, Mehrvarzfar et al. reported CH to be superior to BAG 45S5 [61]. In contrast, Krithika datta et al. reported no significant difference between both the medicaments. Also, antibacterial efficacy was reported to increase with an increase in time [40, 48].

Krithika datta et al. showed 2% CHX gel to be more effective than BAG [48]. However, the present study results revealed that a combination of CH + BAG S53P4 is more effective in reducing the bacterial load than CH + 2% CHX gel. Studies reported to date have not assessed the combined efficacy of CH with BAG over other agents such as chitosan and AGNP.

Regarding CH and CH + 2% CHX gel, the authors of the present study have reported CH + 2% CHX gel to show a better antibacterial reduction. The reason is the interaction of the positive charge of the CHX with the phosphate groups on microbial cell walls, which is negatively charged, thereby altering the osmotic equilibrium of the cell. Eventually, cell wall permeability increases, enabling the CHX molecule to penetrate the bacteria [47]. Previous studies reported similar results, which showed CHX to be superior to CH [48, 62, 63].

Its substantivity, protein-binding and least cytotoxic properties have also been well documented in the literature [64]. Basically CHX is a bis-biguanide, which exhibits a bacteriostatic effect at lower concentration and bactericidal effect at higher concentrations. The optimal antibacterial effect of CHX is achieved at a pH of 5.5 to 7 [18]. The ideal properties of CHX as an intracanal medicament are mainly exhibited due to its alkaline pH [65]. It has various benefits when used as a medicament. It is claimed to neutralize the remnant organic tissue debris in the root canal after chemo-mechanical debridement [66]. It induces an osteo-promotive environment by continuous release of hydroxyl ions, leading to increased osteogenic activity [65]. Its additional benefits include the neutralization of bacterial cell walls, especially the lipopolysaccharide, and also help in better disinfection of the root canal system [67]. The toxic effects on the bacterial cell wall are mainly achieved by the interaction of the positively charged molecule of CHX with the negatively charged bacterial cell wall. There are reports claiming the increased antimicrobial activity of CHX, when combined with CH [68]. Usually, the combination aims at increasing the antimicrobial activity of CH while maintaining its biological and mechanical properties [68]. The literature claims the additive antimicrobial effect is not due to the CHX component alone. But the research claims the adjunctive effect is mainly due to the realse of various byproducts from the CHX, by fragmentation process. As these byproducts tend to have a higher pH, they exhibit both as an antioxidant and pro-oxidant, thereby enhancing its effects.

Savita et al. reported that both agents showed the highest bacterial reduction against *E. faecalis* in case of persistent infections [69]. Literature evidence has also shown that 2% CHX to be more efficient in bacterial reduction than 2% chitosan [70]. The reason might be due to the increased diffusion of CHX into the dentinal tubules [48, 71, 72]. Also, CHX was found to have increased penetration into dentinal tubules compared to chitosan [73].

Perochena et al. demonstrated the addition of chitosan nanoparticles to CH significantly had better antibacterial efficacy than CH when used alone [74]. The reason might be due to the enhanced stability of calcium ion binding to the surface of chitosan nanoparticles. Similar results were also observed in the study by Archana et al. [70].

It has also been reported as being biocompatible and biodegradable, and with suitable antibacterial properties. Furthermore, the chitosan is claimed to have a higher surface area and charge density and react with the bacterial cell surface, eventually destroying the bacterial cells [75]. Chitosan has an immense potential in inhibiting the biofilm formation. Its ant biofilm activity is mainly due to interaction occurring with microbial cellular and sub-cellular components [71]. The major advantage with chitosan is its higher biocompatibility and its lower cytotoxicity making it an ideal material to be used as an intracanal medicament.

Charannya et al. reported a combination of CHX and AGNP showed higher antibacterial activity than individual compounds [76]. The antimicrobial action against bacterial cells might vary depending on the characteristics of nanoparticles, such as contact time, concentration, particle size and surface charge [34].

Contact time for AGNP plays a vital role in eliminating microorganisms from the root canal system. The antibacterial activity is due to the interaction of AGNP with the bacterial cell wall, which causes structural changes leading to the destruction of tissue protein [37]. It also has an increased surface area to volume ratio, which increases the reactivity [77]. The antibacterial property was reported to be enhanced with the smaller particle size of approximately 1–100 nm, increasing the chemical reactivity [37]. AGNP have a broader spectrum antibacterial and anti-biofilm activity and due to their smaller size they are able to penetrate the complex areas of the root canal system.

It is said to be biocompatible when used at a lower concentration [78]. In vitro study data reported the increased oxidative stress, cytotoxicity and pulmonary inflammation with the larger sized AGNP as compared with the smaller ones [79]. There is always a controversial report on the evident cytotoxic nature of these nanoparticles. Indeed, studies have shown that AGNP not be suggested as root canal irrigant [29, 80].

Rodrigues et al. had reported no significant difference between AGNP and CHX. The present study result revealed that a combination of CH with CHX showed reduced antibacterial efficacy compared with CH with a AGNP [33].

The present study results correlated with Afkhami et al., wherein CH with AGNP showed better antibacterial efficacy than CH with CHX [81]. Also, CH with AGNP showed better bacterial reduction when compared to CH alone, which is also similar to our study results.

When the limitations of the present study have to be considered, CFU assessing the *E. faecalis* might not indeed translate a clinical condition. The main limitation of the study is that, it did not employ biofilm analysis rather than study assessed only the planktonic culture. The complex root canal anatomy houses the infected tissue along with the biofilm masses. Ideally, the root canal biofilm is extremely complex and organized. Its almost a difficult task or impossible to duplicate its characteristics in the invitro experimentations. Studies on mono or dual species over simplify the real root canal ecology and might not give a true implication of the clinically achievable results [82]. Hence, future studies are advised to be carried out by considering all these factors.

## Conclusions

Combination with calcium hydroxide showed higher antibacterial efficacy than when used alone. Amongst which calcium hydroxide with bioactive glass showed better antimicrobial efficiency against *E. faecalis* when compared to other combinations of calcium hydroxide.

## Data Availability

The data will be available on reasonable request from the corresponding author.
